# Effects of familial hypercholesterolemia-associated genes on the phenotype of premature myocardial infarction

**DOI:** 10.1186/s12944-019-1042-3

**Published:** 2019-04-11

**Authors:** Chongyou Lee, Yuxia Cui, Junxian Song, Sufang Li, Feng Zhang, Manyan Wu, Long Li, Dan Hu, Hong Chen

**Affiliations:** 1 Department of Cardiology, Peking University People’s Hospital, Xizhimen South Rd. No.11, Xicheng district, Beijing, 100044 China; 2 Beijing Key Laboratory of Early Prediction and Intervention of Acute Myocardial Infarction, Peking University People’s Hospital, Beijing, China; 3 Center for Cardiovascular Translational Research, Peking University People’s Hospital, Beijing, China

**Keywords:** Premature myocardial infarction, Cholesterol metabolism, Familial hypercholesterolemia, Gene mutation

## Abstract

**Background:**

The incidence of premature myocardial infarction (PMI) has gradually increased in recent years. Genetics plays a central role in the development of PMI. Familial hypercholesterolemia (FH) is one of the most common genetic disorders of cholesterol metabolism leading to PMI.

**Objective:**

This study investigated the relationship between FH-associated genes and the phenotype of PMI to clarify the genetic spectrum of PMI diseases.

**Method:**

This study enrolled PMI patients (*n* = 225) and detected the mutations in their FH-associated genes (LDLR, APOB, PCSK9, LDLRAP1) by Sanger sequencing. At the same time, patients free of PMI (non-FH patients, *n* = 56) were enrolled as control, and a logistic regression analysis was used to identify risk factors associated with PMI. The diagnosis of FH was confirmed using “2018 Chinese expert consensus of FH screening and diagnosis” before the prevalence and clinical features of FH were analyzed.

**Results:**

Pathogenic mutations in LDLR, APOB, PCSK9 and LDLRAP1 genes were found in 17 of 225 subjects (7.6%), and all mutations were loss of function (LOF) and heterozygous. The genotype-phenotype relationship of patients carrying FH-associated mutations showed high heterogeneity. The logistic regression analysis showed that the smoking history, obesity and the family history of premature CHD were independent risk factors of PMI. In this study, a total of 19 patients (8.4%) were diagnosed as FH, and the proportion of smoking subjects in FH patients was higher than that in non-FH patients.

**Conclusions:**

FH-associated gene mutations were present in about 7.6% of Chinese patients with PMI. In addition to genetic factors, smoking history, lifestyle and other environmental factors may play a synergistic role in determining the phenotype of PMI.

**Trial registration:**

Essential gene mutation of cholesterol metabolism in patients with premature myocardial infarction. ChiCTR-OCH-12002349.Registered 26 December 2014, http://www.chictr.org.cn/showproj.aspx?proj=7201.

## Introduction

As the most severe type of coronary artery diseases, myocardial infarction (MI) has posed a threat to public health because of its high morbidity and mortality. More than 600,000 people suffer from MI, which leads to 180,000 deaths yearly in China [1, 2]. In recent years, the incidence of premature myocardial infarction (PMI) has gradually increased [3]. It has been shown that genetics plays a central role in the development of PMI, with its heritability estimated at approximately 63% [4]. It was also reported that about 10–15% of PMI cases were caused by essential mutations in genes related to cholesterol metabolism [5]. Familial hypercholesterolemia (FH) is one of the most common genetic disorders of cholesterol metabolism [6], and the mutations in FH-associated genes, such as low-density lipoprotein receptor (LDLR), apolipoprotein B (APOB), proprotein convertase subtilisin/kexin type 9 (PCSK9) and low-density lipoprotein receptor adaptor protein 1 (LDLRAP1), can increase the plasma levels of low-density lipoprotein cholesterol (LDL-C) and lead to PMI.

A previous study carried out by the author of this study showed that the prevalence of FH diagnosed by genetic testing was 4.4% [7]. However, it was also found that not all clinical phenotypes of PMI matched gene mutations. Therefore, this article aimed to investigate the relationship between FH-associated gene and the phenotype of PMI to clarify the genetic spectrum of PMI.

## Materials and methods

### Study population

All MI patients were enrolled at Peking University People’s Hospital between May 1, 2015 and March 31, 2017. MI, including ST-segment elevation MI and non-ST-segment elevation MI, which was defined according to the Third Universal Definition of Myocardial Infarction [8]. PMI patients (age at the first MI onset: males of ≤55 years old, or females of ≤60 years old) were included as the experimental group, while gender matched patients free of PMI (non-PMI, age at the first MI onset: males of ≤55 years old, or females of ≤60 years old) were enrolled as the control group. PMI patients with incomplete clinical data or with no blood samples were excluded. The investigational protocol of this study was approved by the ethics review board of Peking University People’s Hospital and was registered into the Chinese Clinical Trial Register (registration number: ChiCTR-OCH-12002349, registry URL: http://www.chictr.org.cn/showproj.aspx?proj=7201). All subjects provided written informed consent at the time of their enrollment. The investigational protocol was designed in accordance with CONSORT2010.

### Collection of clinical and laboratory data

Data of PMI patients were retrieved from previously published studies to assess the clinical characteristics of PMI [7]. The clinical characteristics of non-PMI patients, including age, sex, body mass index (BMI), and family history of premature coronary heart disease (CHD), were collected via their medical records. Laboratory examination results, such as those of routine blood test and biochemical test carried out during the first 24 h after hospital admission, were also obtained. The severity of CHD was assessed according to the Gensini score system described in a previous study [9]. A family history of premature CHD was defined as males of < 55 years old or females of < 60 years old in the first-degree relatives.

### Diagnostic criteria for FH

FH was diagnosed using “2018 Chinese expert consensus of FH screening and diagnosis” [10]. Adults who met 2 of the following criteria could be diagnosed as FH: (1) untreated LDL-C ≥ 4.7 mmol/L (180 mg/dl); (2) skin/tendon xanthoma or corneal arcus in a person of < 45 years old; (3) A first-degree relative with FH or premature arteriosclerotic cardiovascular disease (ASCVD). In addition, FH was also diagnosed by detecting the pathogenic mutations in LDLR, APOB, PCSK9 and LDLRAP1 genes.

### Collection of blood cell samples

Peripheral venous blood samples were collected after patients were admitted into the hospital and were processed within 30 min of collection. Blood cells was isolated by centrifugation at 3000 rpm/min for 10 min and then transferred into new tubes, which were stored at − 80 °C until use.

### DNA extraction

Genomic DNA was extracted from blood cell samples using a DNeasy Blood Kit (Tianyihuyuan, Beijing, China) according to the manufacturer’s protocol.

### Sequencing and analysis of mutations

The entire exon region of LDLR, PCSK9, and LDLRAP1 genes, as well as the exon 26 of APOB gene (from 100 bp to 200 bp of p.Arg3500) located at the LDL receptor-binding site, were studied by Sanger sequencing using an ABI 3730-XL Genetic Analyzer (ABI, Foster City, CA).

The LOVD database (*https://databases.lovd.nl/shared/genes/*), the NCBI-ClinVar database (*https://www.ncbi.nlm.nih.gov/clinvar/*), and the NCBI-Pubmed database (*https://www.ncbi.nlm.nih.gov/pubmed*) were used to determine whether the sequenced mutations were “pathogenic” or “potentially pathogenic” mutations that have been reported. Polyphen-2 software was used to predict whether a newly found mutation was pathogenic.

### Statistical analysis

SPSS19.0 software was used for analysis. Measurement data with a normal distribution were represented by X ± S and examined using independent samples t-test. Count data were expressed by (RQ) and examined using X^2^ tests. A logistic regression analysis model was used to evaluate the correlation between various risk factors and PMI. *P* < 0.05 indicated significant difference.

## Results

### Genetic phenotypes of PMI patients

A total of 225 PMI patients meeting the inclusion criteria were collected, including 188 males (83.6%) and 37 females (16.4%). 19 non-synonymous variants were identified in these PMI patients, including 12 pathogenic mutations and 7 benign variants (Table 1). Among these variants, 5 pathogenic variants (LDLR c.129G > C, c.1867A > T; PCSK9 c.1792G > A; LDLRAP1 c.65G > C, c.274G > A) and 4 benign variants (LDLR c.928A > T, c.2320G > A; PCSK9 c.517C > T, c.1954A > G) were discovered for the first time.

**Table 1:** Genetic phenotypes of PMI patients

Gene	Function	cDNA position	Protein position	Significance	
LDLR	Missense	c.129G > C	p.Lys43Asn	likely pathogenic	Putative
LDLR	Missense	c.241C > T	p.Arg81Cys	pathogenic	Known
LDLR	Missense	c.292G > A	p.Gly98Ser	pathogenic	Known
LDLR	Missense	c.1525A > G	p.Lys509Glu	pathogenic	Known
LDLR	Missense	c.1691A > G	p.Asn564Ser	pathogenic	Known
LDLR	Missense	c.1691A > G	p.Asn564Ser	pathogenic	Known
LDLR	Missense	c.1867A > T	p.Ile623Phe	likely pathogenic	Putative
LDLR	Missense	c.2054C > T	p.Pro685Leu	pathogenic	Known
LDLR	Missense	c.2054C > T	p.Pro685Leu	pathogenic	Known
LDLR		c.928A > T	p.Ile310Phe	benign	Putative
LDLR		c.1057G > A	p.Glu353Lys	benign	Known
LDLR		c.1171G > A	p.Ala391Thr	benign	Known
LDLR		c.1516G > A	p.Val306Met	benign	Known
LDLR		c.2320G > A	p.Asp774Asn	benign	Putative
PCSK9	Missense	c.277C > T	p.Arg93Cys	pathogenic	Known
PCSK9	Missense	c.277C > T	p.Arg93Cys	pathogenic	Known
PCSK9	Missense	c.277C > T	p.Arg93Cys	pathogenic	Known
PCSK9	Missense	c.277C > T	p.Arg93Cys	pathogenic	Known
PCSK9	Missense	c.277C > T	p.Arg93Cys	pathogenic	Known
PCSK9	Missense	c.277C > T	p.Arg93Cys	pathogenic	Known
PCSK9	Missense	c.1792G > A	p.Ala598Thr	likely pathogenic	Putative
PCSK9		c.517C > T	p.Pro173Ser	benign	Putative
PCSK9		c.1954A > G	p.Asn652Asp	benign	Putative
APOB	Missense	c.10579C > T	p. Arg3527Trp	pathogenic	Known
LDLRAP1	Missense	c.65G > C	p.Trp22Ser	likely pathogenic	Putative
LDLRAP1	Missense	c.274G > A	p.Val92Met	likely pathogenic	Putative

*LDLR* low-density lipoprotein receptor, *APOB* apolipoprotein B, *PCSK9* proprotein convertase subtilisin/kexin type 9, *LDLRAP1* low-density lipoprotein receptor adaptor protein 1

Pathogenic mutations in LDLR, APOB, PCSK9 and LDLRAP1 genes were found in 17 of the 225 subjects (7.6%), and all pathogenic mutations were loss of function (LOF) and heterozygous. However, these mutations also included 7 PCSK9 LOF mutations. In contrast to LOF mutations in LDLR, APOB and LDLRAP1, PCSK9 LOF mutations could increase hepatic LDLR expressions and reduce circulating levels of LDL. One patient carried LDLR(c.129G > C), PCSK9(c.277C > T) and LDLRAP1(c.274G > A) mutations, and all other patients carried a single gene mutation. As shown in a previous study [7], the prevalence of FH was 4.4% in PMI patients diagnosed by genetic testing.

### Clinical phenotypes of PMI patients

Among the 225 PMI patients, their age at MI onset was (46.64 ± 7.21) years old. In addition, the average age at MI onset in 56 non-PMI patients was (73.73 ± 6.97) years old.

Compared to non-PMI patients, the PMI patients had a higher level of LDL-C or body mass index (BMI), and were more likely to have a smoking history and a family history of premature CAD (Table 2). The logistic regression analysis showed that the differentially expressed risk factors were independent predictive factors for patients with PMI (Table 3). Although the genetic factor (a family history of premature CHD) was associated with PMI (OR = 2.840; 95% CI: 1.075–7.503; *P* = 0.035), its impact on PMI was relatively weak, because PMI might also be affected by BMI, smoking and other factors.

**Table 2:** Clinical phenotypes of patients with PMI

	Patients with PMI (*n* = 225)	Non-PMI patients (*n* = 56)	*P* value
Male, n(%)	188 (83.6)	45 (80.4)	0.569
BMI (kg/m^2^)	26.71 ± 3.51	24.58 ± 4.12	0.001
Age of MI onset (years)	46.64 ± 7.21	73.73 ± 6.97	< 0.001
Family history of PCHD, n (%)	49 (21.8)	5 (8.9)	0.029
eGFR (ml/min/1.73m^2^)	96.72 (82.49, 104.27)	78.45 (67.71, 89.32)	< 0.001
LDL-C (mmol/L)	3.63 (2.97, 4.35)	3.29 (2.49, 3.86)	0.005
LVEF (%)	60.93 ± 10.25	60.67 ± 8.75	0.861
Gensini score	54 (34, 79)	58.5 (45.5, 83.5)	0.107
Multivessel lesion, n(%)	176 (78.2)	48 (85.7)	0.212
Smoking, n (%)	153 (68.0)	27 (48.2)	0.006
Hyperlipemia, n (%)	75 (33.3)	15 (26.8)	0.347
Hypertension, n (%)	116 (51.6)	35 (62.5)	0.142
Diabetes, n (%)	83 (36.9)	14 (25.0)	0.094

*PMI* premature myocardial infarction, *BMI* body mass index, *PCHD* premature coronary heart disease, *eGFR* estimated glomerular filtration rate, *LDL-C* low-density lipoprotein cholesterol, *LVEF* left ventricular ejection fraction; All *P* values represented the comparisons between PMI patients and non-PMI patients. Comparisons between groups were performed with the student’s t-test for continuous variables and Chi-square test for categorical variables. *P* < 0.05 was considered statistically significant

**Table 3:** Logistic regression analysis of PMI patients

Risk factors	B	OR	95%CI	P
LDL-C (mmol/L)	0.404	1.498	1.127–1.991	0.005
Family history of PCHD, n (%)	1.044	2.840	1.075–7.503	0.035
BMI (kg/m^2^)	0.172	1.188	1.079–1.307	< 0.001
Smoking, n (%)	0.732	2.080	1.128–3.835	0.019

*PMI* premature myocardial infarction, *LDL-C* low-density lipoprotein cholesterol, *PCHD* premature coronary heart disease, *BMI* body mass index

In this study, PMI patients were divided into 4 groups according to their age (Table 4). The proportions of males and patients with a smoking history were both > 50% in all age groups. However, with the increase in age, both the proportion of males and the proportion of patients with a smoking history decreased. Besides, patients younger than 30 years old had the highest level of LDL-C, highest Gensini scores and the highest incidence with a family history of PCHD.

**Table 4:** Age-related Characteristics of PMI patients

	Age ≤ 30 (*n* = 9)	30 ≤ age < 40 (*n* = 34)	40 ≤ age < 50 (*n* = 105)	50 ≤ age < 60 (*n* = 77)
Male, n(%)	9 (100)	34 (100)	93 (88.6)	52 (67.5)
Age at MI onset (years)	28.33 ± 1.58	37.74 ± 3.02	46.33 ± 2.91	53.57 ± 2.21
BMI (kg/m^2^)	29.17 ± 4.53	28.42 ± 3.90	26.81 ± 3.64	25.52 ± 2.44
Family history of PCHD, n (%)	5 (55.6)	11 (32.4)	17 (16.2)	16 (20.8)
Smoking, n (%)	9 (100)	27 (79.4)	73 (69.5)	44 (57.1)
LDL-C (mmol/L)	3.98 (3.87,5.42)	3.67 (3.18,4.36)	3.61 (2.79,4.34)	3.65 (3.10, 4.40)
LVEF (%)	52.16 ± 11.96	58.92 ± 10.25	60.96 ± 10.56	62.81 ± 9.04
Gensini scores	78 (38,90)	55 (25.25,86.76)	62 (56.35,68.15)	53 (35,78)
Multivessel lesion, n(%)	6 (66.7)	27 (79.4)	84 (80)	59 (76.6)

*PMI* premature myocardial infarction, *BMI* body mass index, *PCHD* premature coronary heart disease, *LDL-C* low-density lipoprotein cholesterol, *LVEF* left ventricular ejection fraction

### Clinical phenotypes of patients carrying mutations

**Fig. 1:**
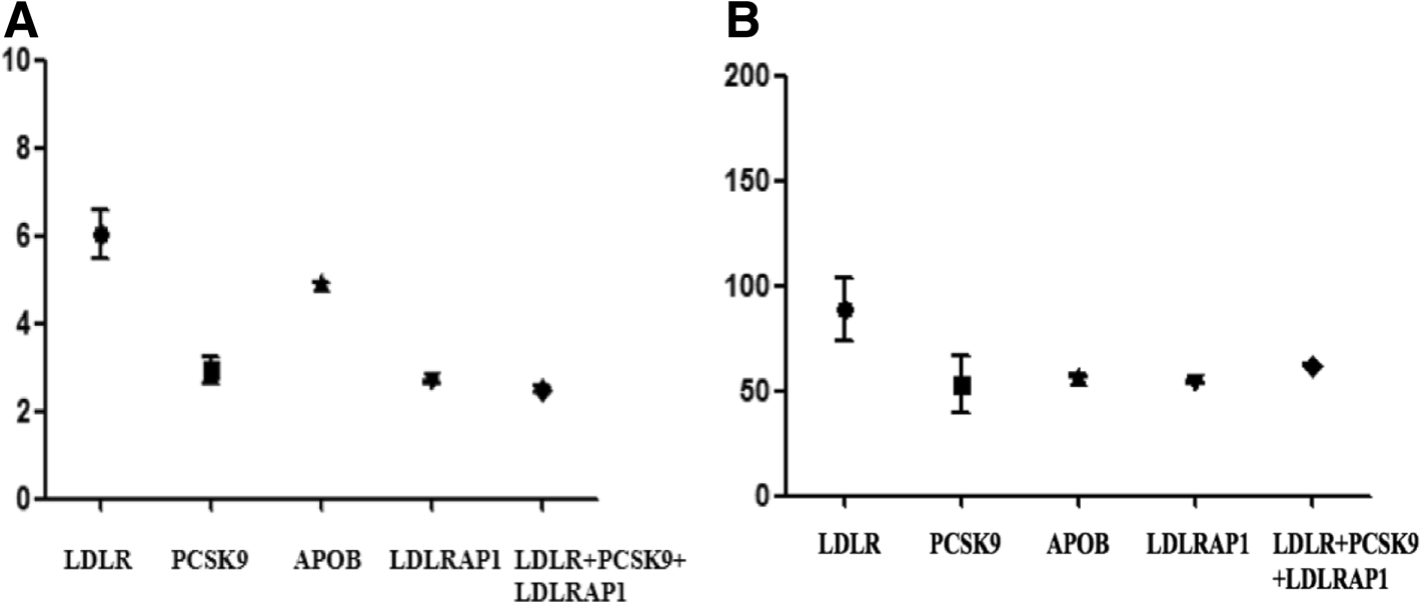
LDL-C levels and Gensini scores of patients carrying mutations. LDLR, low-density lipoprotein receptor; APOB, apolipoprotein B; PCSK9, proprotein convertase subtilisin/kexin type 9; LDLRAP1, low-density lipoprotein receptor adaptor protein 1

Patients carrying mutations in different genes or different mutations of the same gene showed different levels of LDL-C and CHD severity (Table 5). The LDL-C level and Gensini scores were the highest in patients carrying LDLR mutations, followed by patients carrying APOB mutations (Fig. 1).

**Table 5:** Clinical phenotypes of LDLR, APOB, PCSK9 and LDLRAP1 gene mutations

Case	Gene	Function	Exon	Sites of the Mutation	LDL-C (mmol/L)	Gensini Scores
Case1	LDLR	Missense	3	c.241C > T	4.86	40
Case2	LDLR	Missense	3	c.292G > A	3.89	48
Case3	LDLR	Missense	10	c.1525A > G	8.00	54
Case4	LDLR	Missense	11	c.1691A > G	4.35	128
Case5	LDLR	Missense	11	c.1691A > G	7.25	100
Case6	LDLR	Missense	13	c.1867A > T	5.72	104
Case7	LDLR	Missense	14	c.2054C > T	7.74	78
Case8	LDLR	Missense	14	c.2054C > T	6.37	157
Case9	APOB	Missense	26	c.10579C > T	4.93	57
Case10	PCSK9	Missense	2	c.277C > T	3.26	40
Case11	PCSK9	Missense	2	c.277C > T	2.68	40
Case12	PCSK9	Missense	2	c.277C > T	1.66	168
Case13	PCSK9	Missense	2	c.277C > T	3.46	12
Case14	PCSK9	Missense	2	c.277C > T	3.02	65
Case15	PCSK9	Missense	3	c.1792G > A	3.60	117
Case16	LDLRAP1	Missense	1	c.65G > C	2.66	54
Case17	LDLR+PCSK9+LDLRAP1				2.50	62

*LDLR* low-density lipoprotein receptor, *APOB* apolipoprotein B, *PCSK9* proprotein convertase subtilisin/kexin type 9, *LDLRAP1* low-density lipoprotein receptor adaptor protein 1

The LDL-C level of patients carrying PCSK9 LOF mutations ranged from 1.66 mmol/L to 3.60 mmol/L, but the level of LDL-C did not match the severity of CHD. For example, the LDL-C level in Case 12 was only 1.66 mmol/L, but the Gensini score of this patient was the highest among patients carrying PCSK9 LOF mutations (Table 5). Most of these patients had a smoking history, and some of them suffered from diabetes and hypertension.

### Clinical phenotypes of FH patients

Among the 225 PMI patients, 11 (4.9%) patients met at least two of the Chinese criteria for FH diagnosis. Of these patients, 2/11 (1.8%) patients were also diagnosed by genetic testing. At the end, a total of 19 patients (8.4%) were diagnosed as FH. The proportion of smoking subjects in FH patients was higher than that in non-FH patients (Table 6).

**Table 6:** Clinical phenotypes of FH patients

	FH patients (*n* = 19)	Non-FH patients (*n* = 206)	*P* value
Male, n(%)	17 (89.5)	171 (83.0)	0.467
BMI (kg/m^2^)	27.82 ± 5.28	26.60 ± 3.30	0.334
Age at MI onset (years)	46.68 ± 7.97	50.43 ± 7.91	0.050
Family history of PCHD, n (%)	11 (57.9)	38 (18.4)	< 0.001
eGFR (ml/min/1.73m^2^)	93.95 (76.40, 101.71)	97.37 (84.17, 104.48)	0.435
LDL-C (mmol/L)	4.93 (4.80, 6.37)	3.58 (2.95, 4.21)	< 0.001
LVEF (%)	61.31 ± 12.22	60.90 ± 10.08	0.869
Gensini scores	62 (48, 92)	53.5 (32, 77.25)	0.087
Tendon xanthoma/corneal arcus n(%)	0 (0)	0 (0)	1.000
Multivessel lesion, n(%)	15 (78.9)	161 (78.2)	0.936
Smoking, n (%)	17 (89.5)	136 (66.0)	0.036
Hyperlipemia, n (%)	11 (57.9)	64 (31.1)	0.018
Hypertension, n (%)	4 (21.1)	112 (54.4)	0.005
Diabetes, n (%)	7 (36.8)	76 (36.9)	0.996

*FH* familial hypercholesterolemia, *LDL-C* low-density lipoprotein cholesterol, *BMI* body mass index, *LVEF* left ventricular ejection fraction. All *P* values represent comparisons between PMI patients and non-PMI patients. Comparisons between groups were performed with student’s t-test for continuous variables and with Chi-square test for categorical variables. *P* < 0.05 was considered statistically significant

## Discussion

MI places a heavy psychological and the socioeconomic burden on “young” patients because it greatly affects the patients’ ability to work,. The registration study of acute myocardial infarction in China (CAMI) showed that PMI patients accounted for about 27.7% of all MI patients [11]. Because genetic factors play a great role in PMI, the identification of genetic variants conferring susceptibility to PMI is important for the prevention and management of this condition.

In this study, it was observed that 7.6% PMI patients carried FH-associated mutations and the value was higher than that reported in the study by Khera et al., who showed that a familial hypercholesterolemia mutation was present in 36 of 2081 (1.7%) patients with early-onset myocardial infarction [12]. The above difference may be attributed to the different geographical regions and nationalities studied in the two reports [13]. In this study, LDLR gene mutations made up the vast majority of all mutations. However, because all LDLRAP1 mutations are heterozygous, they are hence not pathogenic.

PCSK9 LOF mutations (PCSK9 c.277C > T, c.1792G > A) were also found in this study. In particular, as a mutation previously reported only in the Japanese population, the c.277C > T mutation can decrease the level of LDL-C and reduce the risk of ASCVD [14]. However, the patients in this study did not show lower levels of LDL-C and its reason might be their lifestyle and other environmental factors. In this study, the logistic regression analysis showed that smoking, obesity and family history of premature CHD were independent risk factors for PMI, suggesting that the lifestyle played an important role in the onset of PMI.

Men dominated PMI patients in most studies on PMI, including this study [15, 16], which may be attributed to the misperception that females are ‘protective’ against cardiovascular diseases. Previous studies have also shown that PMI patients were usually cigarette smokers and the proportion of PMI smokers increased over a decreasing age [17, 18], which was consistent with the result of this research. Since patients younger than 30 years were found to have more serious coronary artery lesions, both genetic (family history of PCHD) and environmental (smoking) factors play an important role in the onset of PMI.

The genotype-phenotype relationship of patients with FH-associated mutations showed high heterogeneity. Numerous studies have demonstrated that the carriers of LDLR mutations had the highest levels of LDL-C [19, 20], which was consist with the result of this research that showed the median LDL-C level in carriers of LDLR variants was 5.72 mmol/L and was higher than that in the carriers of other mutations. In addition, the coronary lesions in carriers of LDLR variants were more severe than those in carriers of other mutations. However, patients carrying the same mutation also showed obviously different levels of LDL-C and CAD severity. Such clinical heterogeneity might be attributed to the following reasons. On the one hand, the penetrance of these genes was not 100%; on the other hand, the different diets and lifestyles adopted by different patients might affect their clinical phenotypes.

In this study, the “2018 Chinese expert consensus of FH screening and diagnosis” was used first to diagnose FH. Only 2/11(1.8%) patients with clinical FH carried FH-associated gene mutations and the percentage was lower than that reported in a previous study, suggesting that different diagnostic criteria of FH may lead to different prevalence values of FH. Most studies have also found that 20–60% of subjects with phenotypic FH did not carry a causative mutation in LDLR, APOB, PCSK9 or LDLRAP1 genes, suggesting that phenotypic FH may also be induced by multiple small-effect common variants, mutations in unknown FH-associated genes, or environmental factors [21, 22]. None of the FH patients in this study had Tendon xanthoma/corneal arcus, which was consistent with the results of previous studies [23, 24].

Nevertheless, several limitations in this study need to be addressed. Firstly, the sample size in this study is small and may cause a statistical bias. Secondly, the approach used in this study to annotate missense variants by prediction algorithms may lead to misclassification in some cases, and the predicted variants need to be validated through in vitro functional experiments. Thirdly, only 4 FH-associated common genes were detected in this study, while some other rare FH-associated genes, including APOE and STAP1, were not detected.

In conclusion, FH-associated gene mutations were present in about 7.6% of Chinese PMI patients. In addition to genetic factors, smoking history, lifestyle and other environmental factors may play a synergistic role in determining the phenotype of PMI.

## Abbreviations

APOB: Apolipoprotein B
ASCVD: Arteriosclerotic cardiovascular disease
BMI: Body mass index
CHD: Coronary heart disease
FH: Familial hypercholesterolemia
LDL-C: Low-density lipoprotein cholesterol
LDLR: Low-density lipoprotein receptor
LDLRAP1: Low-density lipoprotein receptor adaptor protein 1
MI: Myocardial infarction
PCSK9: Proprotein convertase subtilisin/kexin type 9
PMI: Premature myocardial infarction
